# Pseudomonas aeruginosa Meningitis: A Case Report and Therapeutic Approach

**DOI:** 10.7759/cureus.75227

**Published:** 2024-12-06

**Authors:** Asmaa Hazim, Yasmine Mimouni, Sarra Saaf, Mustapha Sodki, Fatema Zahra Beniaz

**Affiliations:** 1 Neurology, Cheikh Khalifa International University Hospital, Mohammed VI International University Hospital, Casablanca, MAR; 2 Neurology, Hôpital Cheikh Khalifa Bin Zayed, Casablanca, MAR; 3 Infectious Disease, Faculté de Médecine et de Pharmacie de Casablanca, CHU Ibn Rochd, Casablanca, MAR; 4 Emergency Department, Cheikh Khalifa International University Hospital, Mohammed VI International University Hospital, Casablanca, MAR

**Keywords:** antibiotherapy, asepsia, critical care, meningitis, pseudomonas aeruginosa

## Abstract

We report the case of a 22-year-old mother with no medical history, admitted for gram-negative meningitis, identified as *Pseudomonas aeruginosa*, 15 days after spinal anaesthesia. She was initially treated with dual antibiotic therapy, consisting of ceftazidime (2g three times a day) and amikacin. The first lumbar puncture (LP) performed 10 days approximately after the beginning of the treatment found no bacterial growth on the CSF culture. However, after 21 days of well-conducted treatment, she relapsed, and the *Pseudomonas aeruginosa* was once again detected on the CSF culture. The therapeutic protocol was changed, and she began meropenem and ciprofloxacin, which was pursued for six weeks. She improved clinically and biologically and was discharged after nearly 90 days of hospitalisation.

## Introduction

Iatrogenic meningitis is a rare yet severe and potentially deadly condition. The incidence is estimated to be extremely low, between 0% and 0.04% [1]. Neurosurgical complications have proven to be the main cause of iatrogenic meningitis related to gram-negative bacteremia. However, other factors should be looked for, such as head trauma, spinal anaesthesia or even a history of chronic otitis [2]. A brain neoplasm or haemorrhage is also frequently associated with *Pseudomonas aeruginosa *meningitis [3].

This is the case of a 22-year-old mother who has been diagnosed with *Pseudomonas aeruginosa* meningitis, which occurred following spinal anaesthesia, without presenting any risk factor or any other underlying medical condition.

## Case presentation

We report a case of a 22-year-old mother admitted to our facility for headache, nausea and vomiting 20 days after spinal anaesthesia for a caesarian section. The neurological examination found a well-oriented patient with nuchal rigidity and both Kernig’s and Brudzinski’s signs [4]. 

**Figure 1:**
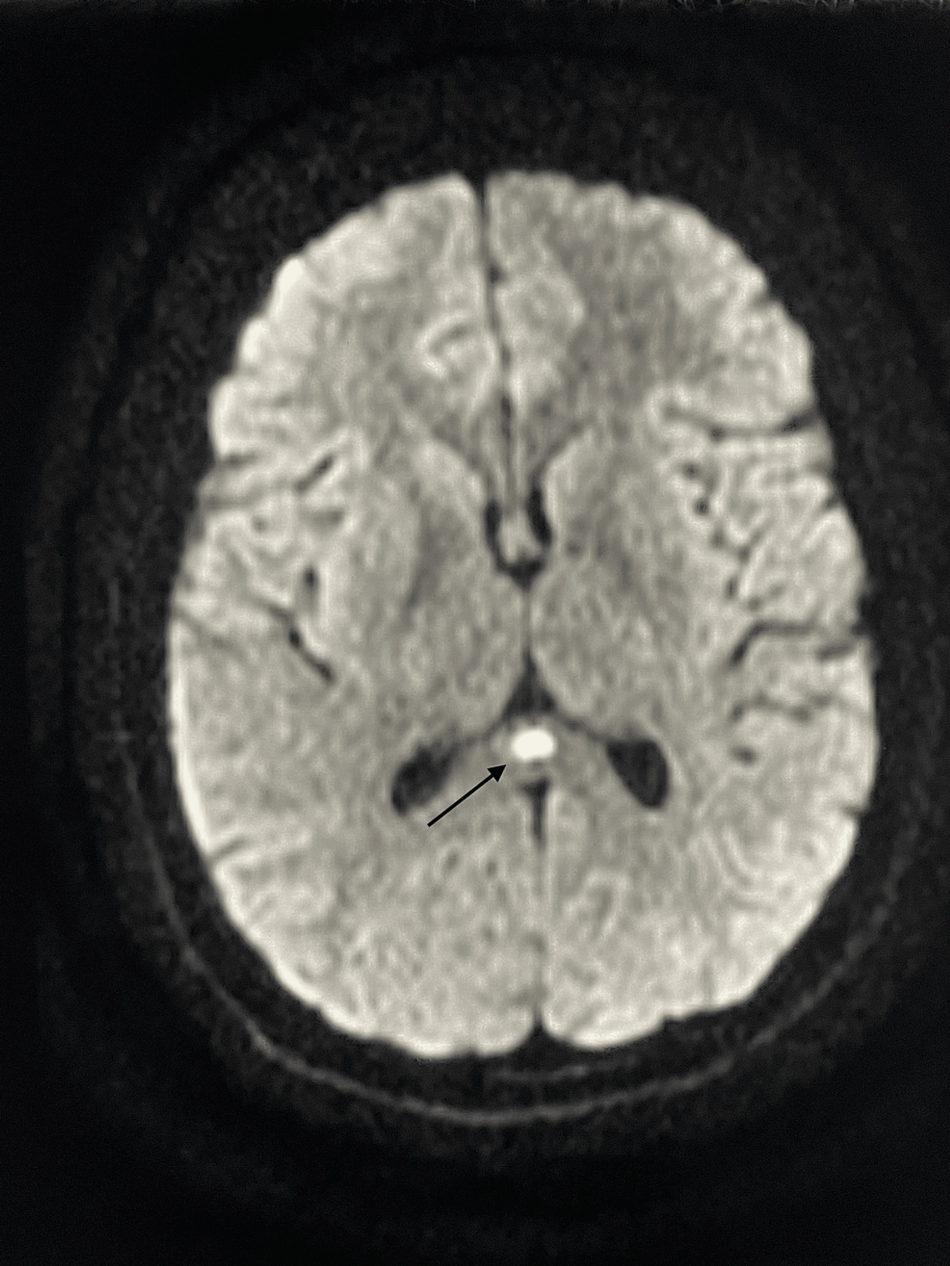
Brain magnetic resonance imaging performed before the antibiotic therapy (axial section, DWI) Diffusion-weighted imaging (DWI) shows a focal, isolated, ovoid, hyperintense signal on the splenium of the corpus callosum (arrow).

The biological parameters were unremarkable. The initial brain MRI revealed an isolated lesion in the splenium of the corpus callosum (Figure 1), the appearance and radiological progression of which have been discussed in greater detail and depth in a previous article [4]. 

However, the initial lumbar puncture (LP) finds an increased level of proteins at 105 mg/dL (reference values: 15-45), a decreased glucose level at 0.12 g/L (reference values: 0.4-0,8) and a lymphocytic pleocytosis at 800 cells/mL, 87% of which are lymphocytes (Table 1). Gram stain found no bacteria. The opening pressure from the LP was normal.

**Table 1:** CSF parameters and antibiotherapy received during the hospitalization

	Day 1	Day 5	Day 10	Day 22	Day 31	Day 48	Day 76	Reference values
White cell count in CSF (cells/mm^3^)	800	790	360	120	30	41	<5	<5
Polymorphonuclear neutrophil (PMN) level in CSF (%)	13	64	60	4	5	0	-	0
Lymphocyte level in CSF (%)	87	36	40	96	95	100	-	0
Culture CSF	Pseudomonas aeruginosa	Pseudomonas aeruginosa	Sterile	Sterile	Pseudomonas aeruginosa	Sterile	Sterile	Sterile
Proteins levels (mg/dL)	105	103	97		35	32	29	15-45
Antibiotherapy	Ceftriaxone + aminoglycosides	-	Ceftazidime + aminoglycosides		Meropenem + fluoroquinolones	-	Fluoroquinolones	-

The patient was put on antibacillary drugs, given the endemic context of tuberculosis in Morocco. Nevertheless, a viral infection couldn't be excluded at this stage. Therefore, an anti-viral medication was initiated. A complementary molecular biology was requested in parallel to the research of the meningitis/encephalitis panel and the BK polymerase chain reaction (PCR) in the CSF, both of which came back negative. The CSF culture found growth of *Pseudomonas aeruginosa* (Table 1) with intrathecal IgG synthesis [4].

Once the bacteria was identified, the patient received a dual antibiotic therapy made of third-generation cephalosporins (6 g per day of ceftriaxone) and aminosides (1 g per day). The antiviral and antibacillary drugs were stopped [4].

**Figure 2:**
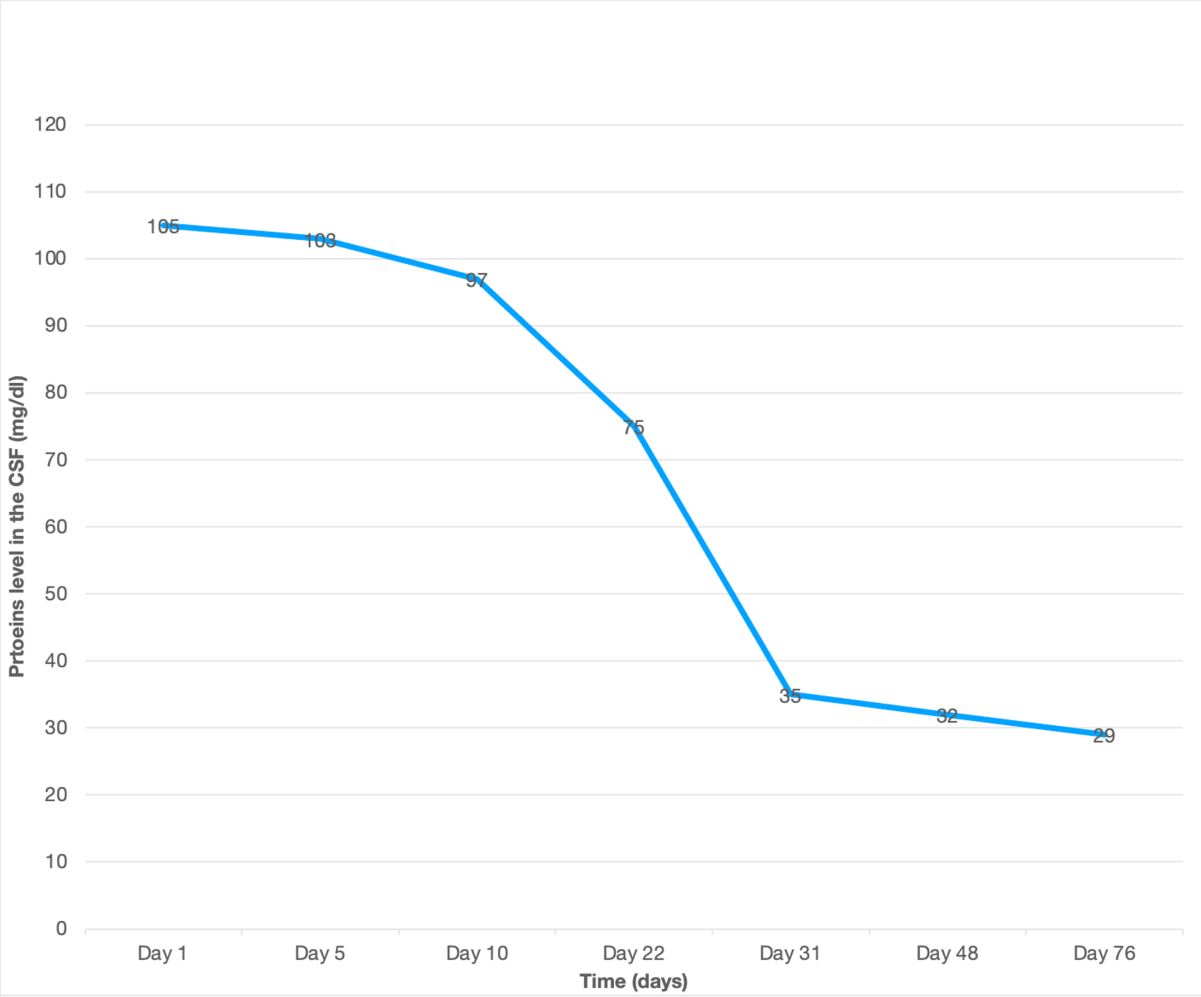
Evolution of the protein levels in the CSF

**Figure 3:**
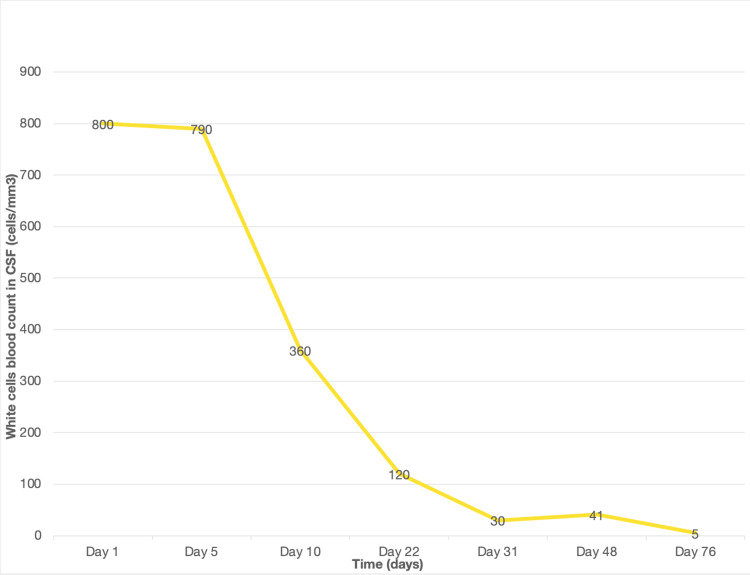
Evolution of the white cell levels in the CSF

After 10 days of treatment, a lumbar punction was performed, and a decrease in white cell count was found even though it was still elevated (Figures 2-3). Following these results, the antibiotic protocol treatment was changed with the replacement of ceftriaxone with ceftazidime (2 g three times a day). However, the culture became sterile with no growth of *Pseudomonas aeruginosa*.

**Figure 4:**
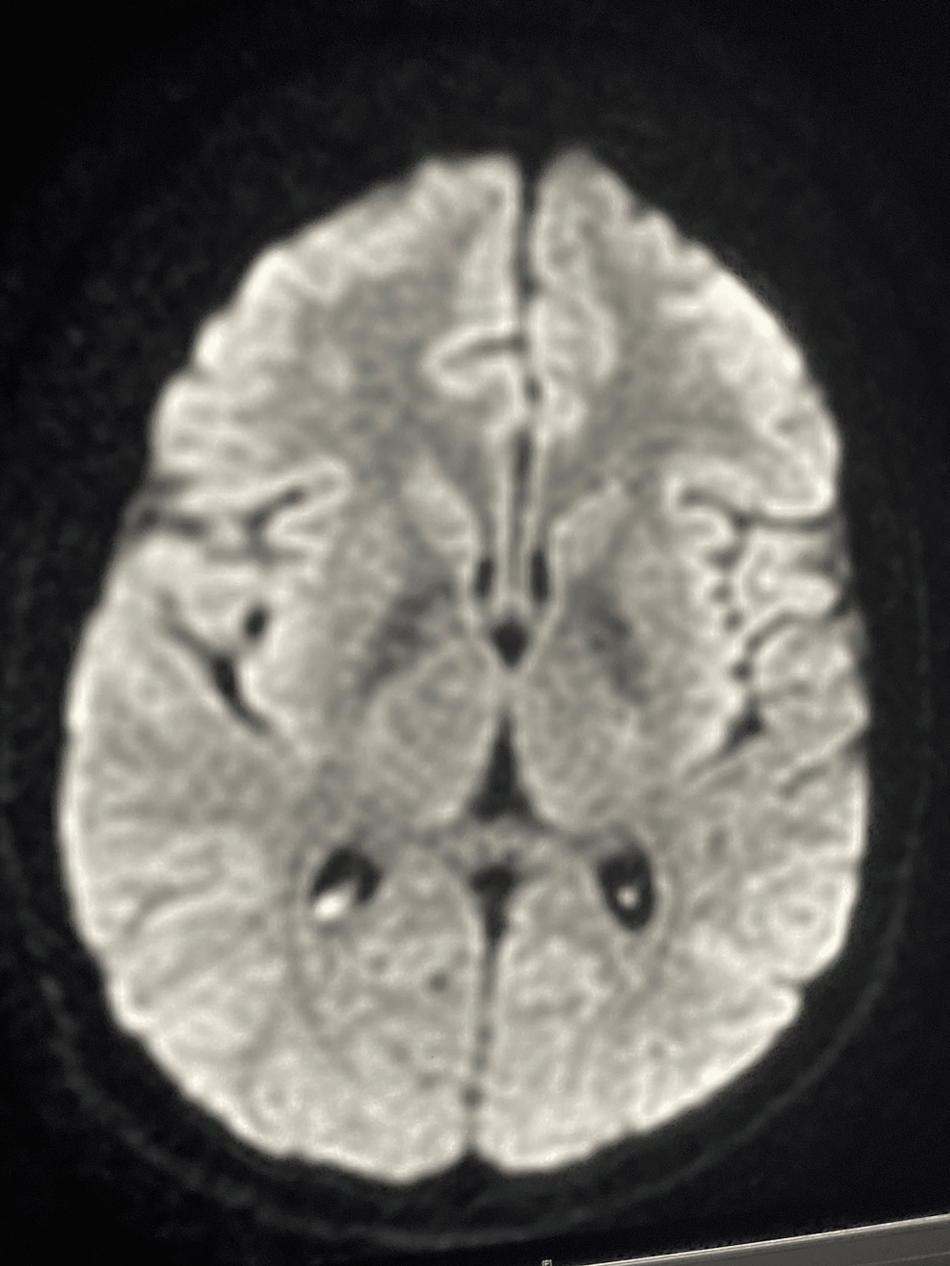
Brain magnetic resonance imaging performed one month after the dual antibiotic therapy (axial section, DWI) Diffusion-weighted imaging (DWI) shows the complete disappearance of the splenial lesion.

After a multidisciplinary consultation meeting, it was decided to maintain the antibiotic therapy for 21 days with regular control of the renal parameters and a good biological improvement with one neutering culture on the follow-up LP, and a progressive decrease of the protein levels and the white blood cell count in the CSF (Figures 2-3). The brain MRI performed on day 21 of the antibiotic therapy showed radiological improvement with the disappearance of the corpus callosum lesion (Figure 4).

Following the 21 days of antibiotherapy, the patient had a fever again with headaches. The CSF analysis found a pleocytosis at 30, with 95% lymphocytes, and the culture grew *Pseudomonas aeruginosa* (Table 1). The susceptibility testing found an intermediate susceptibility for ceftazidime.

The therapeutic protocol was changed again with carbapenem (2 g three times a day) and ciprofloxacin (500 mg twice a day). A preventive anti-epileptic coverage was added. A third brain MRI was performed to rule out any intracranial process or lesion that could explain the clinical deterioration that returned normal.

Although the patient became completely asymptomatic, with a decreased frequency and intensity of the headache, she remained hospitalised with the same dual antibiotic therapy until the CSF parameters were normalised, which occurred on day 76 (Table 1; Figures 2-3). 

After discussing with the infectious diseases team, the carbapenem was stopped, and ciprofloxacin (500 mg three times a day) was kept, as well as the anti-epileptic coverage at the same dosage for a month. 

She was discharged after nearly 90 days of hospitalisation. Clinical follow-up has been favourable so far. The patient is no longer taking antibiotics and is fully asymptomatic. The last CSF analysis was perfectly normal.

## Discussion

*Pseudomonas aeruginosa* is a gram-negative bacillus, usually found in hospitals. Meningeal localisation is not common, and therefore, no consensus on the optimal antibiotherapy protocol treatment has been established.

Nevertheless, the effectiveness of ceftazidime, a broad-spectrum cephalosporin with parenteral administration, has been reviewed and adopted for the treatment of gram-negative meningitis [3,5] due to its antipseudomonal activity but also for its good diffusion through the blood-brain barrier. The study conducted by Fong et al. [5] showed that nine patients treated with ceftazidime alone in IV were cured. Five of these nine patients received prior antibiotics such as carbenicillin and aminoglycosides. The daily dosage of ceftazidime ranges from 2 g/8 hours [5] to 3 g/8 hours [3].

However, the emergence of multidrug-resistant bacteria needs to be taken into account. In the study carried out by Rodriguez-Lucas et al. [3], mortality was higher in patients treated with ceftazidime only. Therefore, other therapeutic combinations were studied. The association of intrathecal and systemic therapy was demonstrated. Aminoglycosides or colistin have been used intrathecally, in association with ceftazidime, with a lower mortality and recurrence rate [3,6]. The recommended duration of treatment ranges from 14 to 28 days [6-8]. 

Our case reports a recurrence after 21 days of treatment with ceftazidime. A biotherapy, including intrathecal antibiotic therapy, could have allowed a complete remission of the patient. A second-line treatment protocol made of carbapenem (meropenem 2 g/8 hours) and ciprofloxacin (500 mg/12 hours) was administered.

Meropenem has been demonstrated to be the only carbapenem backed for the treatment of meningitis. Nevertheless, the Rodriguez-Lucas et al. [3] study showed that the mortality rate of the patients treated by meropenem was close to 12%. 

It has also been reported that Doripenem is another carbapenem proven to be more active against *Pseudomonas aeruginosa* than meropenem. Morelli et al.’s [9] study displayed that the combination of doripenem, rifampicin and colistin, administered intravenously, resulted in a good clinical outcome. 

## Conclusions

The meningeal localisation of the *Pseudomonas aeruginosa* is considered a nosocomial infection associated with a high mortality rate. Broad-spectrum cephalosporins, such as ceftazidime, are considered to be the pillar of therapeutic management.

However, due to the resistant profiles of the bacteria, a systemic and intrathecal combination is recommended.
